# Polyol-Mediated Synthesis of V_2_O_5_–WO_3_/TiO_2_ Catalysts for Low-Temperature Selective Catalytic Reduction with Ammonia

**DOI:** 10.3390/nano12203644

**Published:** 2022-10-18

**Authors:** Min Seong Lee, Yeong Jun Choi, Su-Jeong Bak, Mingyu Son, Jeehoon Shin, Duck Hyun Lee

**Affiliations:** Green Materials and Processes R&D Group, Korea Institute of Industrial Technology, Ulsan 44413, Korea

**Keywords:** polyol-mediated synthesis, NH_3_-selective catalytic reduction, small catalysts

## Abstract

We demonstrated highly efficient selective catalytic reduction catalysts by adopting the polyol process, and the prepared catalysts exhibited a high nitrogen oxide (NO_X_) removal efficiency of 96% at 250 °C. The V_2_O_5_ and WO_3_ catalyst nanoparticles prepared using the polyol process were smaller (~10 nm) than those prepared using the impregnation method (~20 nm), and the small catalyst size enabled an increase in surface area and catalytic acid sites. The NO_X_ removal efficiencies at temperatures between 200 and 250 °C were enhanced by approximately 30% compared to those of the catalysts prepared using the conventional impregnation method. The NH_3_-temperature-programmed desorption and H_2_-temperature-programmed reduction results confirmed that the polyol process produced more surface acid sites at low temperatures and enhanced the redox ability. The in situ Fourier-transform infrared spectra further elucidated the fast absorption of NH_3_ and its reduction with NO and O_2_ on the prepared catalyst surfaces. This study provides an effective approach to synthesizing efficient low-temperature SCR catalysts and may contribute to further studies related to other catalytic systems.

## 1. Introduction

Over recent decades, chemical impurities such as nitrogen oxides (NO_X_), sulfur oxides, carbon oxide (CO), volatile organic compounds, and particulate matter generated from the usage of biomass as a fuel at power plants, boilers, and mobile sources have polluted the atmospheric environment [1,2]. Among these, NO_X_ (NO, NO_2_, and N_2_O) are extremely dangerous, as they cause various environmental issues, such as acid rain, smog, ozone depletion, and even harm to human health [3,4,5,6]. Several processes, such as selective catalytic reduction (SCR), selective noncatalytic reduction (SNCR), nonselective catalytic reduction (NSCR), and photocatalytic degradation of NO_X_ [7,8,9,10,11,12], have been proposed to eliminate NO_X_ [13]. Among them, NH_3_-SCR, which converts NO_X_ in exhaust gas into N_2_ and H_2_O, is the most commercialized technology owing to its 80–100% advanced efficiencies and economic feasibility [14,15]. Several types of composites, including transition metals (Fe, Cu, V, and Mn), are used as SCR catalysts [16,17]. V_2_O_5_–WO_3_/TiO_2_ are representative SCR catalysts, owing to their high catalytic acidity in high temperature ranges of 300–400 °C and lower chemical contamination possibility than other elements [18]. However, V_2_O_5_–WO_3_/TiO_2_ catalysts exhibit low catalytic performance at low temperatures below 300 °C [19,20].

Currently, most coal-fired power plants have adopted a high-dust system with an exhaust gas pretreatment such as installing an economizer at the rear end of the SCR system [21]. Consequently, the SCR catalyst becomes abraded and contaminated by dust and sulfur, reducing the utilization efficiency over time. Therefore, numerous studies have been conducted to develop new catalysts efficient at temperatures below 300 °C [9,22,23,24,25]. The low-temperature catalyst installed at the rear end of the electrostatic precipitator and desulfurization facility enables the realization of the tail-end SCR system and reduces reheating costs [26].

Comprehensive research has been conducted to develop low-temperature SCR catalysts, and Mn- and Cu-based catalysts reportedly exhibit high SCR catalytic performances at low temperatures [16,27,28,29,30,31]. However, they are severely deactivated by sulfur contamination [32]. A mesoporous TiO_2_ shell can improve the resistance of Fe_2_O_3_ catalysts to SO_2_ (Han et al.) [33]. Yu et al. developed a Cu-SSZ-13 zeolite–metal oxide hybrid catalyst with high SO_2_ resistance by forming Zn sulfate [34]. Additional studies on low-temperature catalysts entailed applying functional chemicals to improve the catalytic activity [35,36,37,38,39]. Chae et al. developed a V_2_O_5_–Sb_2_O_3_/TiO_2_ catalyst with a high catalytic performance at temperatures below 300 °C by adding ammonium nitrate, which promoted NO oxidation and rapid SCR mechanism reaction at temperatures below 300 °C [39]. Zhao et al. reported 90% of NO_X_ removal efficiency with the V_2_O_5_/TiO_2_ catalyst at 210 °C by co-doping S and N [40]. These catalysts form O^2–^ active sites, increasing chemisorbed oxygen and NH_3._ Furthermore, Maqbol et al. reported CeO_2_–Sb/V_2_O_5_/TiO_2_ catalysts pretreated with SO_2_ under oxidizing conditions [41] forming sulfate species on the surface and a high NH_3_-desorption and catalytic performance due to cerium (III) sulfate formation. However, previous studies have limitations concerning the complexity of the synthesis process, restricting catalyst composition and limiting their commercial application.

The crystalline quality and morphology of nanomaterials are important in regulating the physicochemical properties of catalysts. In the polyol process, the liquid organic compound, a polyol, including 1,2-diols and ether glycols, acts both as a solvent of the solid precursor and as a reducing agent determining important process characteristics [42,43]: (1) the high boiling point allows synthesis at relatively high temperatures and ensures well-crystallized nanomaterials; (2) the reducing medium protects the as-prepared particles from contamination, as long as they remain in the medium; and (3) the high viscosity of the medium minimizes coalescence and favors a diffusion-controlled regime for particle growth, resulting in controlled structures and morphologies. Thus, the polyol process offers several advantages, including the easy control of nanomaterials, low cost, and verified scalability for industrial applications [42,44].

Herein, we adopted a polyol process to synthesize highly efficient SCR catalysts and compared their catalytic properties with those of a catalyst prepared using the conventional impregnation method. The catalysts synthesized through the polyol process formed small-sized nanoparticles within a short time and, thus, had numerous active sites that could react with NO_X_. The effect of the polyol process on the V_2_O_5_–WO_3_/TiO_2_ catalyst was observed via transmission electron microscopy (TEM), Raman spectroscopy, and Brunauer–Emmett–Teller (BET) analysis. The NOx removal efficiency and N_2_ selectivity of the catalyst were measured to compare the catalytic activities in the low-temperature range of 150–300 °C. NH_3_-temperature-programmed desorption (NH_3_-TPD), H_2_-temperature-programmed reduction (H_2_-TPR), and in situ Fourier-transform infrared (FTIR) spectroscopy were performed to elucidate the enhancement of the catalytic activities.

## 2. Materials and Methods

### 2.1. Materials

Ammonium metavanadate (AMV; NH_4_VO_3_), ammonium metatungstate hydrate (AMT; (NH_4_)_6_H_2_W_12_O_40_ × H_2_O), and oxalic acid (C_2_H_2_O_4_) were purchased from Sigma-Aldrich (St. Louis, MO, USA). Ethylene glycol (C_2_H_6_O_2_) was obtained from Daejung Chemicals (Siheung-si, Korea), and titanium dioxide (TiO_2_) was obtained from NANO Co., Ltd. (Seoul, Korea). All chemicals were of reagent grade and used without further purification.

### 2.2. Preparation of V_2_O_5_–WO_3_/TiO_2_ Catalysts

We prepared 2 wt.% V_2_O_5_–5 wt.% WO_3_/TiO_2_ catalysts using the impregnation and polyol processes. In the impregnation method, AMV (0.128 g, 99.99%) or AMT (0.266 g, 99.99%) was dissolved in 50 mL of deionized water with 0.196 g oxalic acid. TiO_2_ powder (4.650 g, NT-01) was mixed with the prepared solution and stirred for 2 h. The solution was evaporated at 85 °C in an oil bath and placed in an oven at 110 °C for 12 h. The obtained powder was then sintered at 500 °C in a furnace for 5 h under atmospheric pressure. In the polyol process, AMV (0.128 g, 99.99%) or AMT (0.266 g, 99.99%) was dissolved in 100 mL ethylene glycol with 0.196 g oxalic acid. TiO_2_ powder (4.650 g, NT-01) was mixed with the prepared solution and stirred for 2 h. The solution was heated in a microwave (Multiwave 5000; Anton Paar, Graz, Austria) for 10 min at 180 °C. The reacted solution was filtered, washed, and placed in an oven at 110 °C for 12 h. The obtained powder was calcinated at 500 °C in a furnace for 5 h under standard atmospheric pressure. The synthesized catalysts were denoted as IM and P according to the method applied to V_2_O_5_ and WO_3_, respectively. Moreover, V_2_O_5_(P)–WO_3_(IM) and V_2_O_5_(IM)–WO_3_(P) catalysts were impregnated before the polyol process.

### 2.3. Catalyst Characterization

We investigated the morphology of the catalysts using a field emission scanning electron microscope (FESEM; SU8020; Hitachi, Tokyo, Japan) and transmission electron microscope (TEM; JEM-2100F; JEOL Ltd., Tokyo, Japan) at an accelerating voltage of 15.0 kV to understand the effect of the synthesis method on catalysts. The chemical compositions of the catalysts were measured using an X-ray fluorescence spectrometer (XRF; Zetium; Malvern Panalytical, Malvern, UK). The crystallinity and impurities of the catalysts were analyzed by X-ray diffraction (XRD; Ultima IV; Rigaku, Tokyo, Japan), with Cu Kα (λ = 0.15406 nm) radiation in the 2 θ range of 10–90° at a scan rate of 1°/min and Raman spectra (alpha300s; WITec, Ulm, Germany) with a 532 nm laser. The textural properties of the catalysts were analyzed using the BET method (ASAP2020; Micromeritics Instrument Corp., Norcross, GA, USA). NH_3_-TPD was conducted using AutoChem II 2920 (Micromeritics Instrument Corp.). The samples were pretreated at 150 °C with a current of N_2_ for 4 h to remove physiosorbed NH_3_ species and organic matter. NH_3_ was then adsorbed with 10% NH_3_/He gas at 150 °C for 1 h. H_2_-TPR was conducted using the same instruments as NH_3_-TPD, in addition to exposing the catalysts to a current of 10% H_2_/Ar and measuring in the 100–900 °C temperature range.

### 2.4. Catalytic Activity Evaluation

The catalytic performance was evaluated in a fixed-bed reactor under atmospheric pressure. The operating temperature varied from 150 °C to 300 °C, and the reactive gas comprised 300 ppm NO, NH_3_ (NH_3_/NO_X_ = 1.0), and SO_2_, and 5 vol.% of O_2_ with a balance of N_2_ at a total flow rate of 500 sccm. During the evaluation, 0.35 mg of the powdered catalyst (sieved to 40–60 mesh) was tested, yielding a gas hourly space velocity (GHSV) of 60,000 h^−1^. The reactive gas concentration was continuously monitored through FTIR spectroscopy (CX–4000; Gasmet Technologies, Vantaa, Finland) and an O_2_ analyzer (Oxitec 5000; ENOTEC, Marienheide, Germany). The NO_X_ removal efficiency and N_2_ selectivity were calculated according to Equations (1) and (2), respectively.

(1) $${\mathrm{NO}}_{X}\;\mathrm{removal}\;\mathrm{efficiency}\;\left(\%\right)=\frac{{\mathrm{NO}}_{X\;\mathrm{inlet}}-{\mathrm{NO}}_{X\;\mathrm{outlet}}}{{\mathrm{NO}}_{X\;\mathrm{inlet}}}\times 100$$

(2) $$N_{2}\;\mathrm{selectivity}\;\left(\%\right)=\frac{{\mathrm{NO}}_{\;\mathrm{inlet}}-{\mathrm{NO}}_{\;\mathrm{outlet}}-{\mathrm{NO}}_{2\;\mathrm{outlet}\;}-{\;N}_{2}O_{\mathrm{outlet}}}{{\mathrm{NO}}_{\;\mathrm{inlet}}-{\mathrm{NO}}_{\;\mathrm{outlet}}}\times 100$$

### 2.5. In Situ FTIR Measurement

In situ FTIR spectra of all samples were measured using an FTIR spectrometer (VERTEX 70v FTIR; Bruker, Billerica, MA, USA) [45] under operating conditions and accumulated 16 scans with a resolution of 4 cm^−1^ in the range of 4000–400 cm^−1^. The gas mixture of NH_3_ (500 ppm), NO (500 ppm), and O_2_ (5 vol.%) with N_2_ was used for in situ FTIR, and the flow rate was 0.3 L/min.

## 3. Results and Discussion

**Figure 1:**
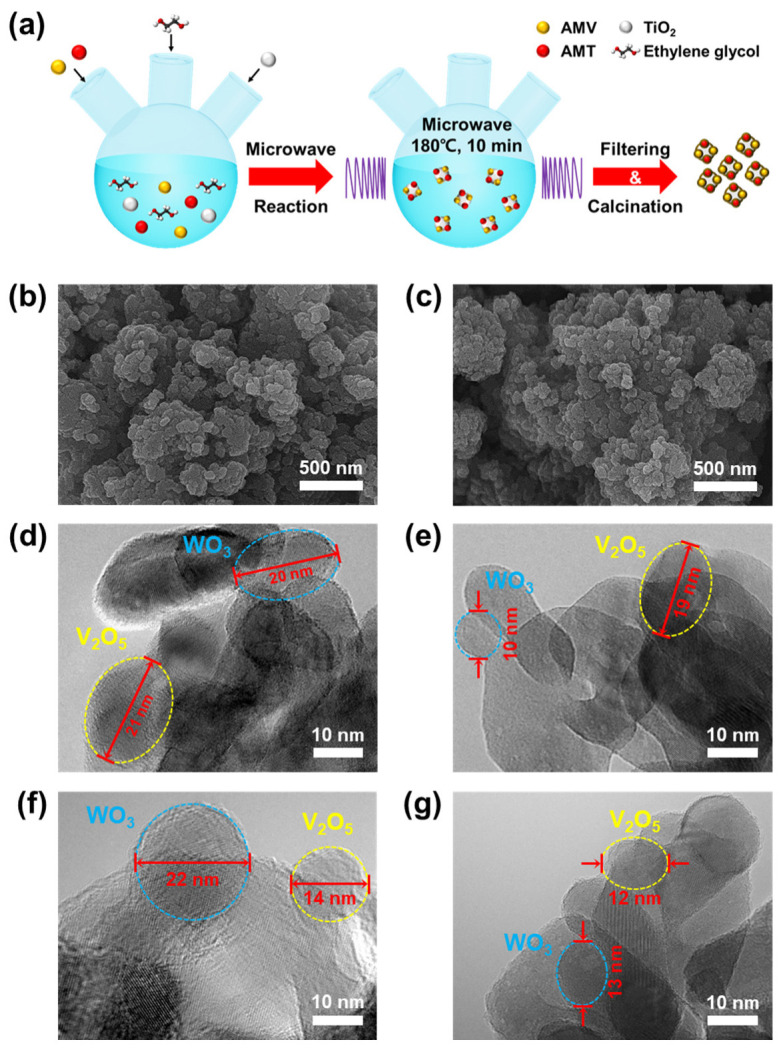
(**a**) Schematic of the polyol process on V_2_O_5_–WO_3_/TiO_2_ catalysts using a microwave at 180 °C for 10 min. Field emission scanning electron microscope images of (**b**) V_2_O_5_(IM)–WO_3_(IM) and (**c**) V_2_O_5_(P)–WO_3_(P). Transmission electron microscope images of (**d**) V_2_O_5_(IM)–WO_3_(IM), (**e**) V_2_O_5_(IM)–WO_3_(P), (**f**) V_2_O_5_(P)–WO_3_(IM), and (**g**) V_2_O_5_(P)–WO_3_(P).

Figure 1a illustrates the formation of vanadium and tungsten oxide nanoparticles on titania with short nucleation and controlled particle growth during the polyol process (V_2_O_5_(P)–WO_3_(P)). We controlled the reaction between the V, W precursor, and ethylene glycol under microwave irradiation at 180 °C for 10 min in an enclosed chamber. The optimized V_2_O_5_–WO_3_/TiO_2_ nanoparticles were obtained using multifunctional microwave equipment. The samples were filtered from the unreacted precursor and ethylene glycol and dried in an oven at 110 °C. Ethylene glycol acts as a stabilizer to limit particle growth and prevent agglomeration. Finally, we obtained green-colored samples with vanadium glycolate and tungsten glycolate. After calcination at 500 °C, the catalysts were transformed into V_2_O_5_–WO_3_ nanoparticles with a yellow color. This polyol process is a facile synthesis process ideal for processing very fine powders with high purity, high crystallinity, good reproducibility, narrow particle size distribution, uniformity, and high reactivity. The overall reactions of vanadium and tungsten are given as Equations (3) and (4), respectively [46].

NH_4_VO_3_ + C_2_H_6_O_2_ ⇒ N_2_ + VO(CH_2_O)_2_ + H_2_O ⇒ V_2_O_5_ (3)

(NH_4_)_6_H_2_W_12_O_40_ × H_2_O + C_2_H_6_O_2_ ⇒ N_2_ + WO(CH_2_O)_2_ + H_2_O ⇒ WO_3_ (4)

Table 1 shows the V_2_O_5_, WO_3_, TiO_2_, and SO_3_ weight fractions of the catalysts. The weight fractions synthesized using the polyol process and impregnation method were similar, except that SO_3_^–^ was present in TiO_2_.

### 3.1. Catalyst Characterization

FE-SEM and TEM were used to compare morphologies of the V_2_O_5_ and WO_3_ nanoparticles synthesized using the impregnation and polyol process, respectively (Figure 1b–g). Figure 1b,c show FE-SEM images of V_2_O_5_(IM)–WO_3_(IM) and V_2_O_5_(P)–WO_3_(P), respectively. The clusters of both catalysts had similar particle sizes and shapes with diameters of approximately 20–30 nm, such as those of titania. Therefore, V_2_O_5_ and WO_3_ nanoparticles are difficult to distinguish from the TiO_2_ particles. In contrast, Figure 1d–g show the distinct V_2_O_5_ and WO_3_ nanoparticles through TEM and diffraction patterns analysis of the samples. V_2_O_5_(IM)–WO_3_(IM), V_2_O_5_(IM)–WO_3_(P), V_2_O_5_(P)–WO_3_(IM), and V_2_O_5_(P)–WO_3_(P) have V_2_O_5_/WO_3_ particle sizes of 21 nm/20 nm, 14 nm/22 nm, 10 nm/19 nm, and 13 nm/12 nm, respectively (Figure S1). The catalyst particle size is very important, because the active area that determines the performance of the catalyst is very important, and these results demonstrate that the polyol process formed smaller V_2_O_5_ and WO_3_ particles than the impregnation method, with up to 55% reduction in particle size.

**Figure 2:**
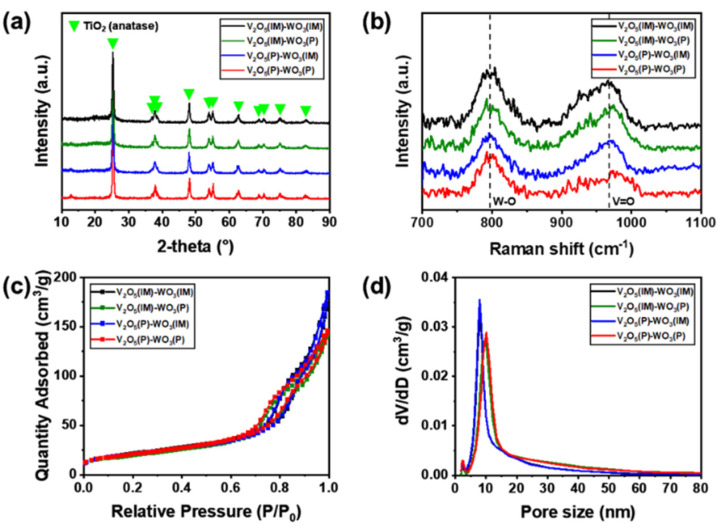
(**a**) X-ray diffraction patterns. (**b**) Raman spectra in the range of 1100 cm^−1^ to 700 cm^−1^. (**c**) N_2_ adsorption−desorption isotherms, and (**d**) Barrett–Joyner–Halenda (BJH) pore size distribution curves of V_2_O_5_(IM)–WO_3_(IM), V_2_O_5_(IM)–WO_3_(P), V_2_O_5_(P)–WO_3_(IM), and V_2_O_5_(P)–WO_3_(P).

The crystalline structure and phase purity of the V_2_O_5_–WO_3_/TiO_2_ catalysts were measured by XRD analysis and Raman spectroscopy. The XRD results showed the anatase phase of TiO_2_ at 25.36°, 37.05°, 37.91°, 38.67°, 48.16°, 54.05°, 55.20°, 62.87°, 68.98°, 70.48°, 75.30°, and 82.93° in all catalysts (Figure 2a). However, the V_2_O_5_ and WO_3_ phases were not observed in any of the catalysts, because the peak positions of V_2_O_5_ and WO_3_ were very similar to those of the anatase phase, and low contents of 2 wt.% V_2_O_5_ and 5 wt.% WO_3_ were uniformly dispersed on TiO_2_ support. Raman spectroscopy was used to understand the crystalline structure and particle size of V_2_O_5_–WO_3_/TiO_2_ catalysts. The Raman spectra of all catalysts contained TiO_2_ anatase peaks at 144.7, 197.3, 401.5, 518.5, and 639.1 cm^–1^ (Figure S2). Figure 2b shows the structure of vanadium and tungsten oxides in the range of 700–1100 cm^–1^. The states of the vanadium and tungsten species on the surface of the catalysts play a crucial role in the SCR catalytic action [47]. The Raman signal at 988.7 cm^–1^ could be attributed to the V–O vibration of crystalline vanadium oxide and at 800.5 cm^–1^ to the W–O–W stretching of octahedrally coordinated W units. V_2_O_5_(IM)–WO_3_(IM) exhibited higher Raman signals than V_2_O_5_(P)–WO_3_(P) at 988.7 cm^–1^ and 800.5 cm^–1^ (Figure 2b), indicating that the impregnation method formed large-sized particles of vanadium and tungsten oxides with high crystallinity, whereas the polyol method formed small-sized particles with low crystallinity. Furthermore, the textural details are listed in Figure 2c,d and Table 2 with the nitrogen adsorption–desorption measurements. All catalysts had similar isotherm plots, corresponding to the H3-type hysteresis loop with a mesoporous structure (Figure 2c). In contrast, the specific surface area, pore volume, and pore size were the highest in the order of V_2_O_5_(P)–WO_3_(P), V_2_O_5_(IM)–WO_3_(P), V_2_O_5_(P)–WO_3_(IM), and V_2_O_5_(IM)–WO_3_(IM), due to the effect of size on vanadium oxide and tungsten oxide particles (Table 2). V_2_O_5_(P)–WO_3_(P) and V_2_O_5_(IM)–WO_3_(P) with the polyol process applied to tungsten oxides had a higher pore size of 14.90 and 14.77 nm, respectively, than V_2_O_5_(P)–WO_3_(IM) (11.68 nm) and V_2_O_5_(IM)–WO_3_(IM) (11.01 nm) (Figure 2d) because of the atomization of the WO_3_ nanoparticles with a content of 5 wt.%, which is a relatively large portion of V_2_O_5_–WO_3_/TiO_2_ than V_2_O_5_ nanoparticles.

### 3.2. Evaluation of Catalytic Activity

In the general NH_3_-SCR process, NO_X_ is converted to nitrogen and water through the reduction reaction of the NH_3_ and NO_X_ on catalysts (Equations (5)–(8)) [48].

4NO + 4NH_3_ + O_2_ ⇒ 4N_2_ + 6H_2_O (5)

NO + NO_2_ +2NH_3_ ⇒ 2N_2_ + 3H_2_O (6)

2NO_2_ + 4NH_3_ + O_2_ ⇒ 3N_2_ + 6H_2_O (7)

6NO_2_ + 8NH3 ⇒ 7N_2_ + 12H_2_O (8)

**Figure 3:**
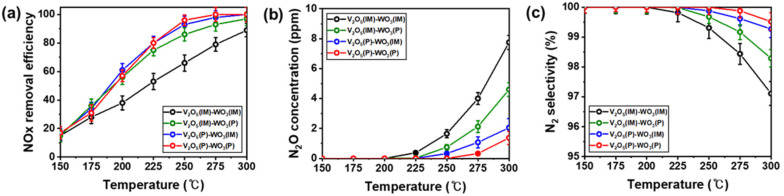
(**a**) Nitrogen oxide (NO_X_) removal efficiency. (**b**) N_2_O concentration and (**c**) N_2_ selectivity of V_2_O_5_(IM)–WO_3_(IM), V_2_O_5_(IM)–WO_3_(P), V_2_O_5_(P)–WO_3_(IM), and V_2_O_5_(P)–WO_3_(P). Reaction conditions: [NO] = [NH_3_] = [SO_2_] = 300 ppm, [O_2_] = 5 vol.%, N_2_ as a balance, and [GHSV] = 60,000 h^−1^.

The SCR catalysts efficiently and selectively reduce NO_X_ to N_2_. The NO_X_ removal efficiency of the V_2_O_5_(P)–WO_3_(P), V_2_O_5_(P)–WO_3_(IM), and V_2_O_5_(IM)–WO_3_(P) catalysts was higher than that of V_2_O_5_(IM)–WO_3_(IM) at 150–300 °C (Figure 3a). At 250 °C, the NO_X_ removal efficiencies of V_2_O_5_(P)–WO_3_(P), V_2_O_5_(P)–WO_3_(IM), and V_2_O_5_(IM)–WO_3_(P) were 96%, 93%, and 86%, respectively, whereas that of V_2_O_5_(IM)–WO_3_(IM) was the lowest at 66%, and the high NO_X_ removal efficiencies of V_2_O_5_(P)–WO_3_(P) were stable for 4 h of the durability test (Figure S3). Based on these results, we found that adjusting the polyol process for the V_2_O_5_–WO_3_/TiO_2_ catalysts increases the specific surface area, leading to enhanced reactions sites for V_2_O_5_ and WO_3_. In particular, the polyol process for V_2_O_5_ nanoparticles was more critical to NO_X_ removal efficiency than WO_3_ nanoparticles, because V_2_O_5_ as the main catalyst is more active than WO_3_. In contrast, all catalysts, including those from the polyol process, exhibited low catalytic activity at 150 °C, demonstrating that vanadium oxide was ineffective, and ammonium sulfate (NH_4_HSO_4_) or ammonium bisulfate ((NH_4_)_2_SO_4_) were easily formed on the catalysts by reacting with sulfur dioxide, unreacted ammonia, and water, blocking the most active sites at temperatures below 150 °C.

Figure 3b,c illustrate the N_2_O concentration and N_2_ selectivity, respectively. Trace amounts of N_2_O in all catalysts were produced at temperatures over 250 °C. N_2_O produced from SCR side reactions is a secondary pollutant, which is important for determining the reaction accuracy. V_2_O_5_(IM)–WO_3_(IM) produced N_2_O at 225 °C, and the amount was relatively large. In contrast, V_2_O_5_–WO_3_/TiO_2_ catalysts formed using the polyol process showed lower N_2_O concentrations than those using the impregnation method, particularly V_2_O_5_(P)–WO_3_(P), which had the lowest N_2_O concentration of 1.375 ppm at 300 °C. According to the N_2_O concentrations, N_2_ selectivity of V_2_O_5_(P)–WO_3_(P), V_2_O_5_(P)–WO_3_(IM), V_2_O_5_(IM)–WO_3_(P), and V_2_O_5_(IM)–WO_3_(IM) reached 99.52%, 99.29%, 98.29%, and 97.11% at 300 °C, respectively.

### 3.3. NH_3_-TPD and H_2_-TPR Analyses

**Figure 4:**
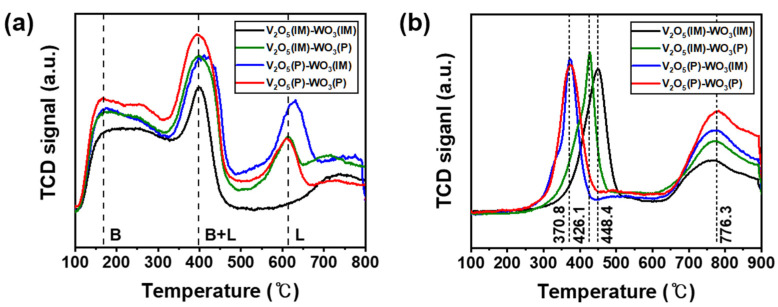
(**a**) NH_3_-temperature-programmed desorption (NH_3_-TPD) profiles and (**b**) H_2_-temperature-programmed reduction (H_2_-TPR) profiles of V_2_O_5_(IM)–WO_3_(IM), V_2_O_5_(IM)–WO_3_(P), V_2_O_5_(P)–WO_3_(IM), and V_2_O_5_(P)–WO_3_(P). B and L indicate Bronsted and Lewis acid sites, respectively.

We further explained the effect of the polyol process on the catalytic performance of the V_2_O_5_–WO_3_/TiO_2_ catalysts using NH_3_-TPD and H_2_-TPR analyses (Figure 4). The NH_3_-TPD results for V_2_O_5_(IM)–WO_3_(IM), V_2_O_5_(IM)–WO_3_(P), V_2_O_5_(P)–WO_3_(IM), and V_2_O_5_(P)–WO_3_(P) were observed at 100–800 °C, which is important for the content and strength of the surface acidic sites on the prepared catalysts (Figure 4a). All curves showed three distinct NH_3_ desorption peaks at 100–200 °C, 300–500 °C, and above 500 °C, indicating weakly, intermediately, and strongly adsorbed NH_3_ related to Bronsted and Lewis acid sites with different intensities, respectively [49,50]. Generally, the adsorbed NH_3_ exists as NH_4_^+^ ions and coordinated NH_3_ when bonded to Bronsted acid sites and Lewis acid sites, respectively. In addition, the concentration of desorbed NH_3_ indicates the adsorption capability of the catalysts. The desorbed NH_3_ concentration for V_2_O_5_(IM)–WO_3_(IM), V_2_O_5_(IM)–WO_3_(P), V_2_O_5_(P)–WO_3_(IM), and V_2_O_5_(P)–WO_3_(P) was 32.86, 51.98, 57.10, and 54.50 cm^3^/g, respectively, in the NH_3_-TPD profile (Table 3). These results indicate that the catalysts from the polyol process have a larger amount of desorbed NH_3_ than those from the impregnation method, because the polyol process induces a large specific surface area and provides various sites for bonding with NH_3_. Particularly, V_2_O_5_(P)–WO_3_(P) showed higher thermal conductivity detector (TCD) signals belonging to Bronsted acid sites in the temperature range of 100–500 °C than V_2_O_5_(IM)–WO_3_(P) and V_2_O_5_(P)–WO_3_(IM), suggesting the explanation for the high catalytic performance of V_2_O_5_(P)–WO_3_(P).

Moreover, we identified the mechanism by which the polyol process affected the redox performance of the catalysts in NH_3_–SCR. The redox performances of V_2_O_5_(IM)–WO_3_(IM), V_2_O_5_(IM)–WO_3_(P), V_2_O_5_(P)–WO_3_(IM), and V_2_O_5_(P)–WO_3_(P) in the temperature range of 100–900 °C by H_2_–TPR analysis are illustrated in Figure 4b and Table 3. The V_2_O_5_(IM)–WO_3_(IM) has three apparent peaks centered at 413.5, 449.5, and 771.0 °C, indicating the co-reduction of V^5+^ to V^3+^ corresponding to the surface vanadium species, reduction of W^6+^ to W^4+^, and reduction of W^4+^ to W^0^ in tungsten oxide, respectively. In contrast, the reduction peaks of V_2_O_5_(IM)–WO_3_(P), V_2_O_5_(P)–WO_3_(IM), and V_2_O_5_(P)–WO_3_(P), indicating V^5+^ to V^3+^ co-reduction and W^6+^ to W^4+^ reduction, shifted to lower temperatures at 400.9 °C/426.3 °C, 340.5 °C/373.7 °C, and 336.3 °C/373.7 °C, respectively, because the increased specific surface area of V_2_O_5_ and WO_3_ promoted the release of lattice oxygen to reduce vanadium and tungsten species, thereby reducing a large amount of hydrogen. Particularly, catalysts that apply polyol to vanadium oxides as active catalysts exhibited remarkable shift changes and reduced a large amount of hydrogen at low temperatures. Therefore, V_2_O_5_(P)–WO_3_(IM) and V_2_O_5_(P)–WO_3_(P) have superior reducing ability, which is one of the reasons for their high NO_X_ removal efficiencies at temperatures below 300 °C.

### 3.4. In Situ FTIR Measurement

**Figure 5:**
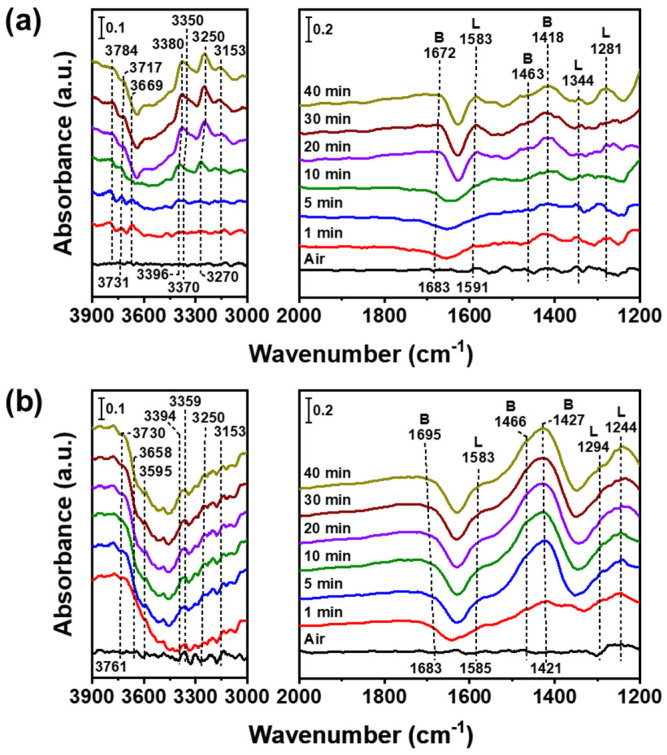
In situ Fourier−transform infrared spectra of ammonia adsorption depending on the reaction time over (**a**) V_2_O_5_(IM)–WO_3_(IM) and (**b**) V_2_O_5_(P)–WO_3_(P) at 200 °C. Conditions: [NH_3_] = 500 ppm (when used) and N_2_ as the balance.

In situ FTIR analysis elucidates the formation and transformation of adsorbed species on the surface of a catalyst, providing information such as the activation capacity of the catalysts or the reaction mechanism between catalysts and reactive gases. Figure 5 illustrates the in situ FTIR spectra of the adsorbed species on the surfaces of V_2_O_5_(IM)–WO_3_(IM) and V_2_O_5_(P)–WO_3_(P) derived from NH_3_ gas at 200 °C. After introducing NH_3_ gas at 200 °C, V_2_O_5_(P) –WO_3_(P) catalysts reacted with NH_3_, and they were mainly covered by coordinated NH_3_ bound to the Lewis acid sites (1244, 1294, 1583, 3153, 3250, 3359, and 3394 cm^−1^) and ionic NH_4_^+^ bound to the Bronsted acid sites (1427, 1466, and 1695 cm^−1^) in 5 min, whereas V_2_O_5_(IM)–WO_3_(IM) reacted with NH_3_ for 20 min [46]. The intensities of the Lewis and Bronsted acid sites were greater in V_2_O_5_(P)–WO_3_(P) than in V_2_O_5_(IM)–WO_3_(IM), demonstrating that the catalysts produced by the polyol process were smaller in size, providing more adsorption sites for ammonia. The FTIR spectra of the adsorbed species on the surfaces of V_2_O_5_(IM)–WO3(P) and V_2_O_5_(P)–WO_3_(IM) were also observed under NH_3_ gas at 200 °C (Figure S4). The catalysts showed intermediate catalytic activities between V_2_O_5_(P)–WO_3_(P) and V_2_O_5_(IM)–WO_3_(IM). The higher specific surface area of the active catalysts (Table 2) provided more Lewis and Bronsted acid sites, resulting in an increase in NH_3_ binding to the catalyst surfaces.

**Figure 6:**
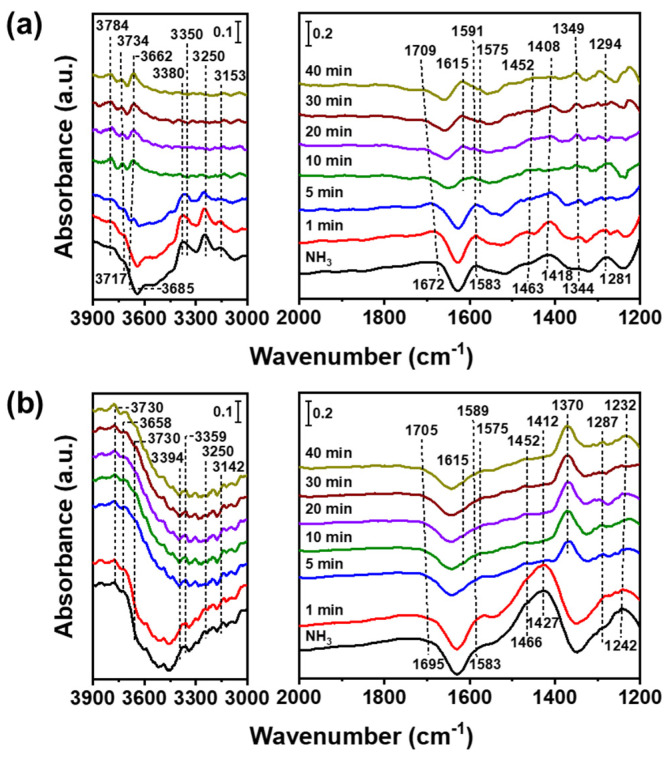
In situ Fourier-transform infrared spectra of nitrogen oxide and oxygen reacted with pre-adsorbed ammonia over **(a)** V_2_O_5_(IM)–WO_3_(IM) and **(b)** V_2_O_5_(P)–WO_3_(P) at 200 °C. Conditions: [NO] = 500 ppm (when used), [O_2_] = 5 vol.% (when used), and N_2_ as the balance.

Figure 6 shows in situ FTIR spectra of NOx and oxygen reacted with pre-adsorbed ammonia over V_2_O_5_(IM)–WO_3_(IM) and V_2_O_5_(P)–WO_3_(P) at 200 °C. V_2_O_5_(P)–WO_3_(P) was primarily covered by coordinated NH_3_ bound to the Lewis acid sites (1232, 1287, 1589, 3142, 3250, 3359, and 3394 cm^–1^) and ionic NH_4_^+^ bound to the Bronsted acid sites (1412, 1452, and 1705 cm^–1^). The adsorbed Lewis and Bronsted acid sites gradually decreased by selectively reducing NO gas, and their reduction was evident in 5 min for V_2_O_5_(P)–WO_3_(P) and 10 min for the V_2_O_5_(IM)–WO_3_(IM) catalysts. The in situ FTIR spectra of V_2_O_5_(IM)–WO3(P) and V_2_O_5_(P)–WO_3_(IM) were also observed under NO and O_2_ gas with pre-adsorbed NH_3_ at 200 °C (Figure S5). The catalysts showed an intermediate reduction time between V_2_O_5_(P)–WO_3_(P) and V_2_O_5_(IM)–WO_3_(IM). V_2_O_5_(P)–WO_3_(P) catalysts showed that the Bronsted acid site disappeared before the Lewis acid site, indicating that the adsorption site corresponds to the Bronsted acid sites, and NO and O_2_ were first bonded. Therefore, the excellent catalytic activity of V_2_O_5_(P)–WO_3_(P) was confirmed when the adsorbed ammonia reacted with NO and O_2_.

## 4. Conclusions

In this study, we explored a facile synthetic process to obtain highly efficient SCR catalysts by adopting a polyol process, and the prepared catalyst demonstrated high NOx removal efficiency of 96% at 250 °C. The V_2_O_5_ and WO_3_ catalyst nanoparticles prepared using the polyol process were smaller (~10 nm) than those prepared using the impregnation method (~20 nm). The small catalyst size enabled an increase in the surface area and catalytic acid sites. At temperatures between 200 and 250 °C, the NOx removal efficiencies were enhanced by approximately 30% compared to the catalysts prepared using the conventional impregnation method. The NH_3_-TPD results demonstrated that the polyol process provided more surface acid sites generated at low temperatures. H_2_-TPR revealed the enhanced redox ability and reducing characteristics of the catalysts, which promoted a rapid SCR reaction. The in situ FTIR spectra elucidated the fast absorption of NH3 and its reduction with NO and O_2_ on the prepared catalyst surfaces at low temperatures. This study provided an effective approach to synthesizing efficient low-temperature SCR catalysts and may contribute to further studies related to other catalytic systems.

## Figures and Tables

**Table 1:** X-ray fluorescence analysis of V_2_O_5_(IM)–WO_3_(IM), V_2_O_5_(IM)–WO_3_(P), V_2_O_5_(P)–WO_3_(IM), and V_2_O_5_(P)–WO_3_(P).

Sample	TiO_2_	V_2_O_5_	WO_3_	SO_3_
V_2_O_5_(IM)–WO_3_(IM)	92.33	1.93	5.02	0.72
V_2_O_5_(IM)–WO_3_(P)	92.43	2.02	4.89	0.66
V_2_O_5_(P)–WO_3_(IM)	92.29	1.91	5.08	0.72
V_2_O_5_(P)–WO_3_(P)	92.44	1.88	4.97	0.71

**Table 2:** Brunauer–Emmet–Teller (BET) results of V_2_O_5_(IM)–WO_3_(IM), V_2_O_5_(IM)–WO_3_(P), V_2_O_5_(P)–WO_3_(IM), and V_2_O_5_(P)–WO_3_(P).

Sample	S_BET_ (m^2^/g)	Pore Volume (cm^3^/g)	Pore Size (nm)
V_2_O_5_(IM)–WO_3_(IM)	71.33	0.22	11.01
V_2_O_5_(IM)–WO_3_(P)	74.23	0.28	14.77
V_2_O_5_(P)–WO_3_(IM)	75.67	0.22	11.68
V_2_O_5_(P)–WO_3_(P)	75.83	0.28	14.90

**Table 3:** NH_3_-temperature-programmed desorption (NH_3_-TPD) and H_2_-temperature-programmed reduction (H_2_-TPR) integral intensity of V_2_O_5_(IM)–WO_3_(IM), V_2_O_5_(IM)–WO_3_(P), V_2_O_5_(P)–WO_3_(IM), and V_2_O_5_(P)–WO_3_(P).

Sample	S_BET_ (m^2^/g)	Pore Volume (cm^3^/g)
V_2_O_5_(IM)–WO_3_(IM)	71.33	0.22
V_2_O_5_(IM)–WO_3_(P)	74.23	0.28
V_2_O_5_(P)–WO_3_(IM)	75.67	0.22
V_2_O_5_(P)–WO_3_(P)	75.83	0.28

## Data Availability

Data are contained within the article.

## Supplementary Materials

The following supporting information can be downloaded at: https://www.mdpi.com/article/10.3390/nano12203644/s1, Figure S1: Transmission electron microscope (TEM) images and selected area electron diffraction (SAED) patterns of (a) V_2_O_5_(IM)–WO_3_(IM), (b) V_2_O_5_(IM)–WO_3_(P), (c) V_2_O_5_(P)–WO_3_(IM), and (d) V_2_O_5_(P)–WO_3_(P). Figure S2: Raman spectra of V_2_O_5_(IM)–WO_3_(IM), V_2_O_5_(IM)–WO_3_(P), V_2_O_5_(P)–WO_3_(IM), and V_2_O_5_(P)–WO_3_(P). Figure S3: NO_X_ removal efficiency of V_2_O_5_(P)–WO_3_(P) measured for 4 h at 250 °C. Figure S4: In situ Fourier-transform infrared spectra of ammonia adsorption, depending on the reaction time over (a) V_2_O_5_(IM)–WO_3_(P) and (b) V_2_O_5_(P)–WO_3_(IM) at 200 °C. Figure S5: In situ Fourier-transform infrared spectra of NO and O_2_ reacted with pre-adsorbed NH_3_ over (a) V_2_O_5_(IM)–WO_3_(P) and (b) V_2_O_5_(P)–WO_3_(IM) at 200 °C.
