# Supplementary material for: Polyol-Mediated Synthesis of V2O5–WO3/TiO2 Catalysts for Low-Temperature Selective Catalytic Reduction with Ammonia
Source: Nanomaterials (Basel). 2022 Oct 18;12(20):3644. doi: 10.3390/nano12203644 (PMC9610785; doi:10.3390/nano12203644)
Supplement: Supplementary file 1 [file nanomaterials-12-03644-s001.zip › nanomaterials-1975681-supplementary.pdf]

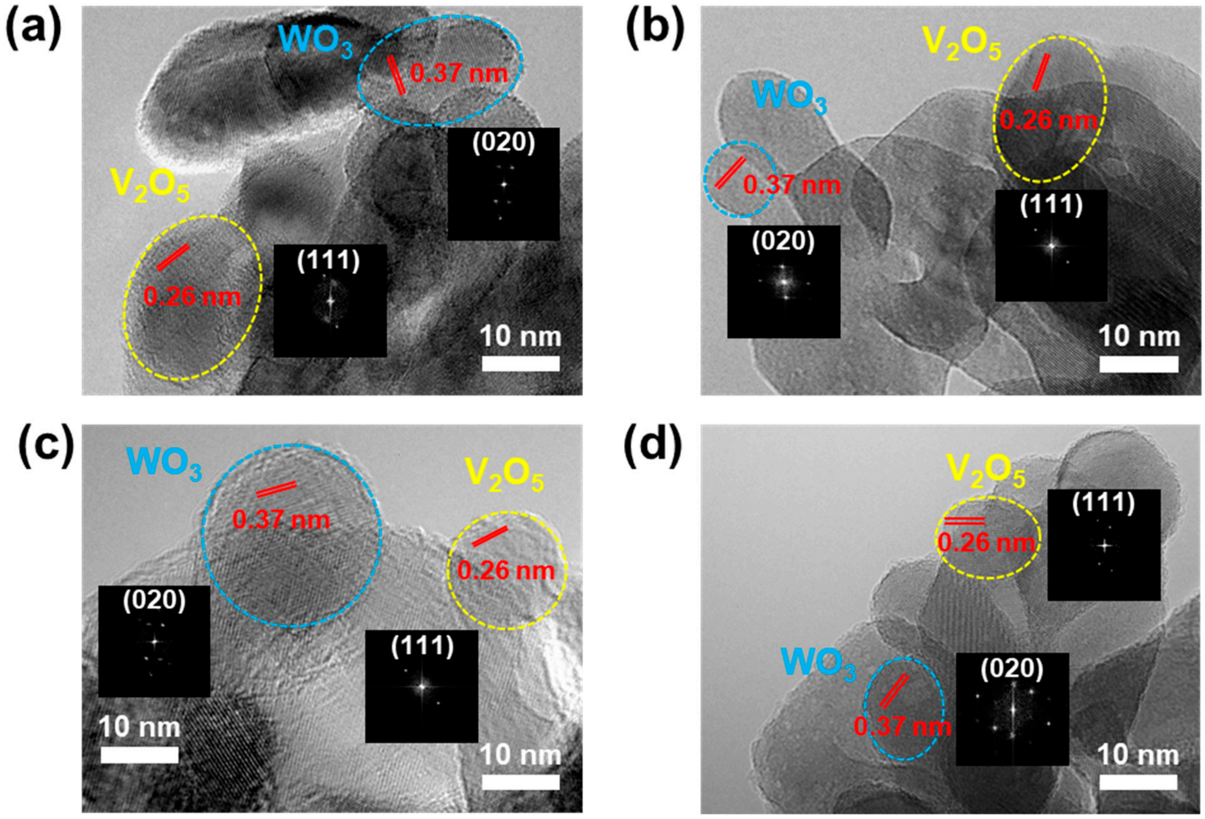

Figure S1. Transmission electron microscope (TEM) images and selected area electron diffraction (SAED) patterns of (**a**) V_2O_5(IM)–WO_3(IM), (**b**) V_2O_5(IM)–WO_3(P), (**c**) V_2O_5(P)–WO_3(IM), and (**d**) V_2O_5(P)–WO_3(P).

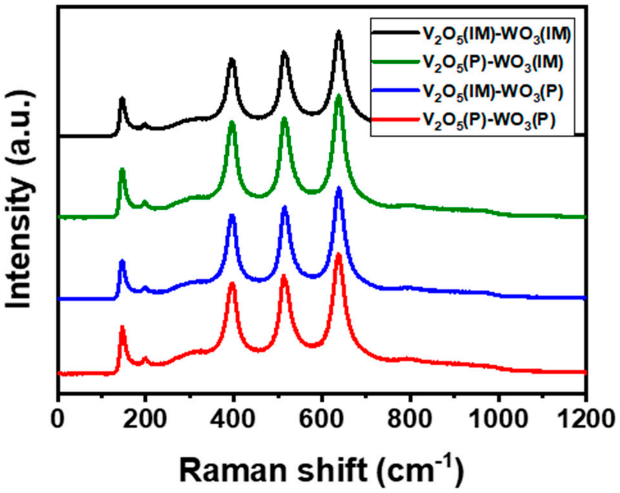

Figure S2. Raman spectra of V_2O_5(IM)–WO_3(IM), V_2O_5(IM)–WO_3(P), V_2O_5(P)–WO_3(IM), and V_2O_5(P)–WO_3(P).

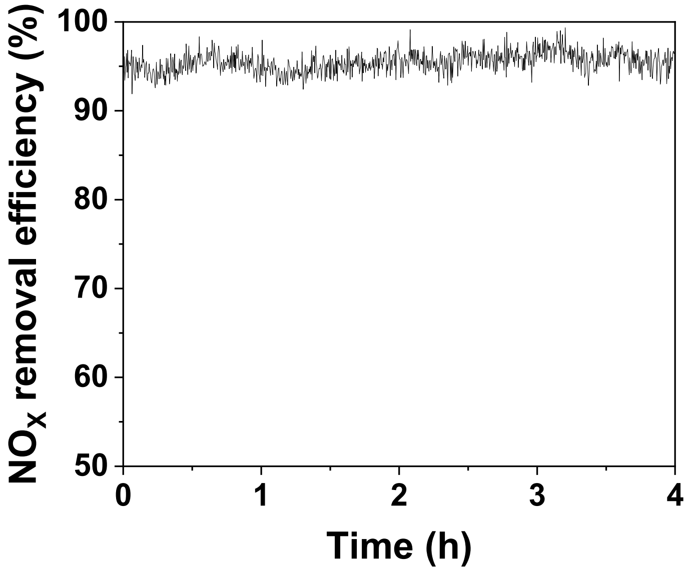

Figure S3. NO_X removal efficiency of V_2O_5(P)–WO_3(P) measured for 4h at 250 °C. Reaction conditions: [NO] = [NH_3] = [SO_2] = 300 ppm, [O_2] = 5 vol.%, N_2 as balance, and [GHSV] = 60,000 h^{-1}.

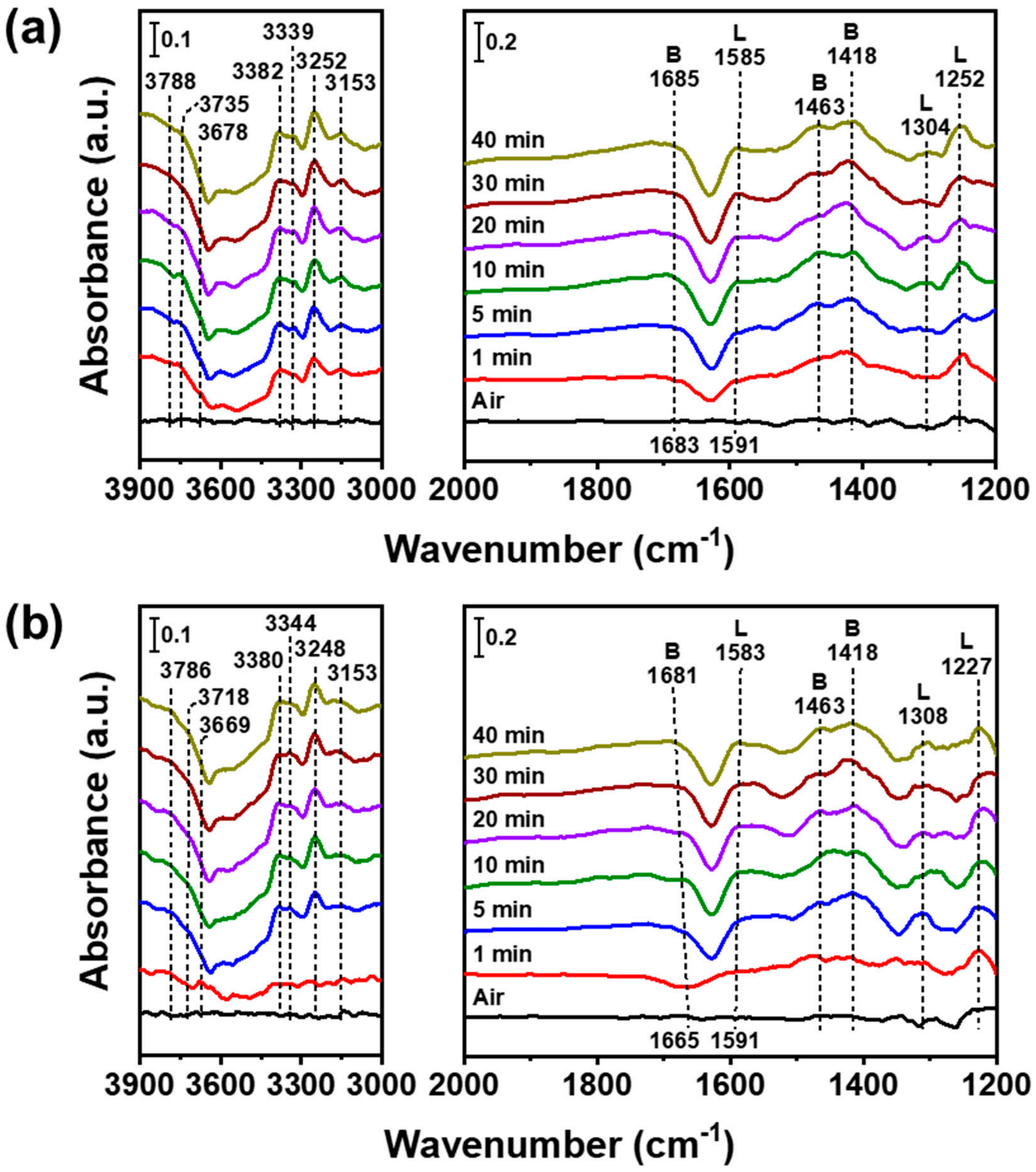

Figure S4. *In situ* Fourier-transform infrared spectra of ammonia adsorption depending on reaction time over (**a**) V_2O_5(IM)–WO_3(P) and (**b**) V_2O_5(P)–WO_3(IM) at 200 °C. Conditions: [NH_3] = 500 ppm (when used) and N_2 as balance.

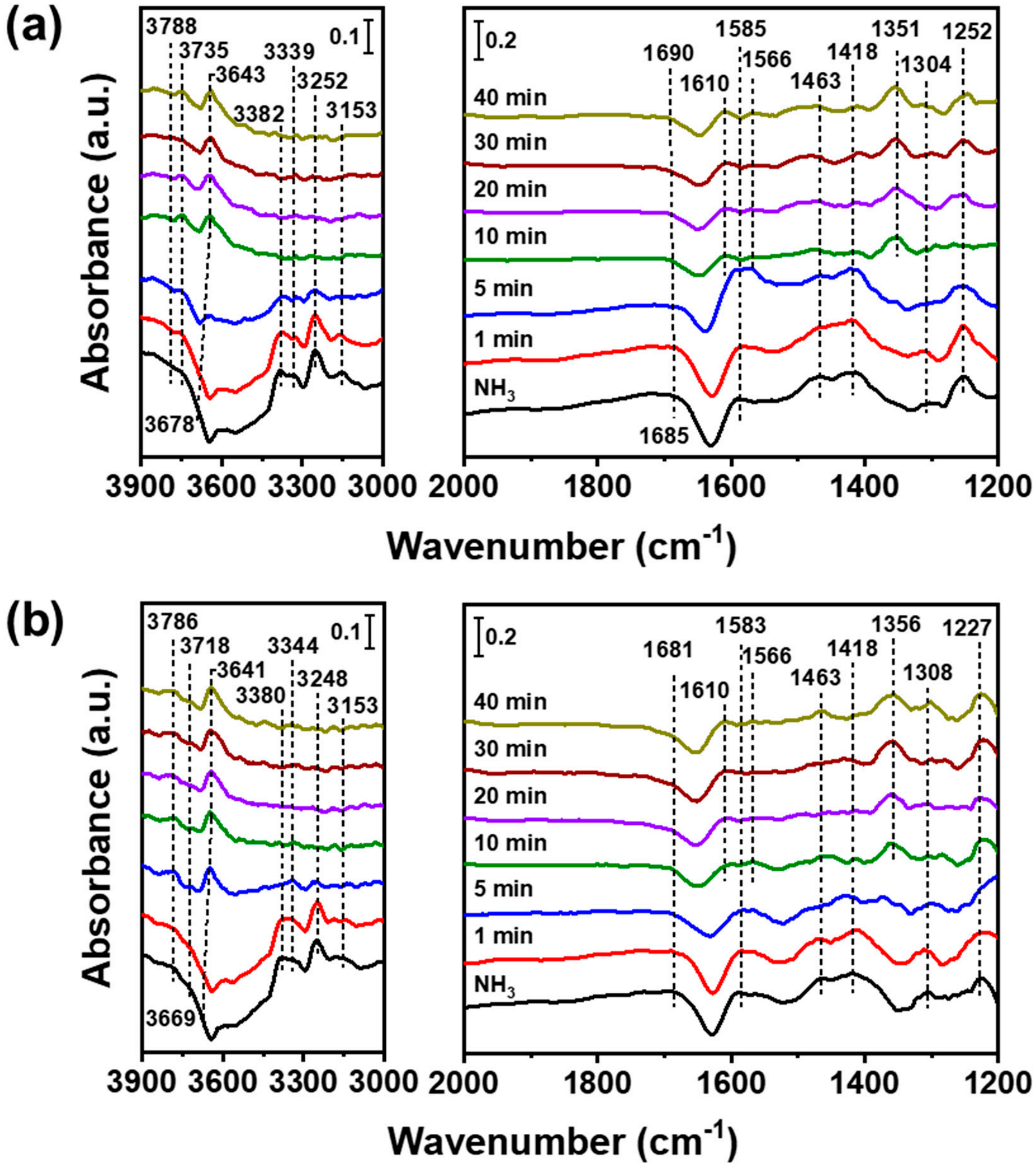

Figure S5. *In situ* Fourier-transform infrared spectra of NO and O_2 reacted with pre-adsorbed NH_3 over (**a**) V_2O_5(IM)–WO_3(P) and (**b**) V_2O_5(P)–WO_3(IM) at 200 °C. Conditions: [NO] = 500 ppm (when used), [O_2] = 5 vol.% (when used), and N_2 as balance.
